# Glycerin-Induced Conformational Changes in *Bombyx mori* Silk Fibroin Film Monitored by ^13^C CP/MAS NMR and ^1^H DQMAS NMR

**DOI:** 10.3390/ijms17091517

**Published:** 2016-09-09

**Authors:** Tetsuo Asakura, Masanori Endo, Misaki Hirayama, Hiroki Arai, Akihiro Aoki, Yugo Tasei

**Affiliations:** Department of Biotechnology, Tokyo University of Agriculture and Technology, Koganei, Tokyo 184-8488, Japan; Kah-vetophs@agate.plala.or.jp (M.E.); misaki.h.0822@gmail.com (M.H.); h-arai@elleair-product.com (H.A.); a_aoki@cc.tuat.ac.jp (A.A.); fv0177@go.tuat.ac.jp (Y.T.)

**Keywords:** *Bombyx mori*, silk fibroin, glycerin, solid state NMR

## Abstract

In order to improve the stiff and brittle characteristics of pure *Bombyx mori* (*B. mori*) silk fibroin (SF) film in the dry state, glycerin (Glyc) has been used as a plasticizer. However, there have been very limited studies on the structural characterization of the Glyc-blended SF film. In this study, ^13^C Cross Polarization/Magic Angle Spinning nuclear magnetic resonance (CP/MAS NMR) was used to monitor the conformational changes in the films by changing the Glyc concentration. The presence of only 5 wt % Glyc in the film induced a significant conformational change in SF where Silk I* (repeated type II β-turn and no α-helix) newly appeared. Upon further increase in Glyc concentration, the percentage of Silk I* increased linearly up to 9 wt % Glyc and then tended to be almost constant (30%). This value (30%) was the same as the fraction of Ala residue within the Silk I* form out of all Ala residues of SF present in *B. mori* mature silkworm. The ^1^H DQMAS NMR spectra of Glyc-blended SF films confirmed the appearance of Silk I* in the Glyc-blended SF film. A structural model of Glyc-SF complex including the Silk I* form was proposed with the guidance of the Molecular Dynamics (MD) simulation using ^1^H–^1^H distance constraints obtained from the ^1^H Double-Quantum Magic Angle Spinning (DQMAS) NMR spectra.

## 1. Introduction

Silk fibroin (SF) from *Bombyx mori* (*B. mori*) is a well-known and highly prized material for textiles. Recently, SF has also been used as a promising biomaterial because of the combination of high strength and toughness together with excellent biocompatibility [1,2,3,4,5]. However, in order to produce effective biomaterials, it is important to improve the shortcomings of SF. For example, SF film tends to become stiff and brittle in the dry state over time, exhibiting high tensile strength but low elongation [6]. In addition, although alcohols such as methanol have been widely used for the treatment of water-soluble SF, methanol induces further stiffness and reduces the biodegradability of SF [1,3,7]. These shortcomings hinder extensive use of SF in biomaterials.

Glycerin (Glyc), a well-known moisturizing agent and plasticizer, has been used to improve the SF properties. Kawahara et al. [8] reported an improvement in the properties of SF film by immersing it in a 10% Glyc aqueous solution. More detailed studies of the improvement of the mechanical properties of the SF films by blending with Glyc were reported by Lu et al. [9]. They showed that Glyc-blended SF films were significantly softer in the dry states, and therefore Glyc should be one of the candidates to overcome the stiffness problem. Pei et al. [10] reported that Glyc induced SF crystallization in the lyophilization process, thereby providing freeze-dried scaffolds with water stability. Compared with salt-leached and methanol-annealed SF scaffolds, the films became softer and enhanced the degradation of the SF scaffold.

It is important to characterize the structure of the Glyc-blend SF films in detail in order to facilitate the widespread use of biomaterials, but only few studies have been reported thus far. Noticeable conformational changes of SF films caused by mixing with Glyc were observed by Fourier transform infrared spectroscopy (FTIR), X-ray diffraction (XRD) and differential scanning calorimetry (DSC) [9,10]. Nuclear magnetic resonance (NMR) gave detailed pictures of the structure and dynamics of SF using both solid and solution state measurements [11,12]. The ^13^C and ^1^H conformation-dependent NMR chemical shifts provided information on the local conformations of amino acid residues and the fraction of each conformation when several conformations were present. Both empirical and quantum chemical studies were reported to use these conformation-dependent chemical shifts for structural determination of proteins and protein-ligand interactions [13,14,15,16,17,18,19]. Several solid-state NMR techniques were developed to determine the structure of peptides, polypeptides and proteins, including the SF structure [11,20,21,22,23].

In this paper, the Glyc-induced conformational changes in Glyc-blended SF film were monitored by ^13^C Cross Polarization/Magic Angle Spinning (CP/MAS) NMR using conformation-dependent NMR chemical shifts and peak deconvolution. In addition, ^1^H Double-Quantum Magic Angle Spinning (DQMAS) NMR [12,24,25,26,27,28,29,30,31,32] was used to confirm the appearance of Silk I* in the Glyc-blended SF film. A structural model of Glyc-SF complex was proposed using Molecular Dynamics (MD) simulation on the basis of the information obtained from ^1^H DQMAS NMR on the ^1^H–^1^H inter-atomic distances in the Glyc-SF complex having the Silk I* structure.

## 2. Results and Discussion

### 2.1. ^13^C Cross Polarization/Magic Angle Spinning Nuclear Magnetic Resonance (CP/MAS NMR) Spectra of Silk Fibroin (SF) and Glycerin (Glyc)-Blended SF Films

**Figure 1:**
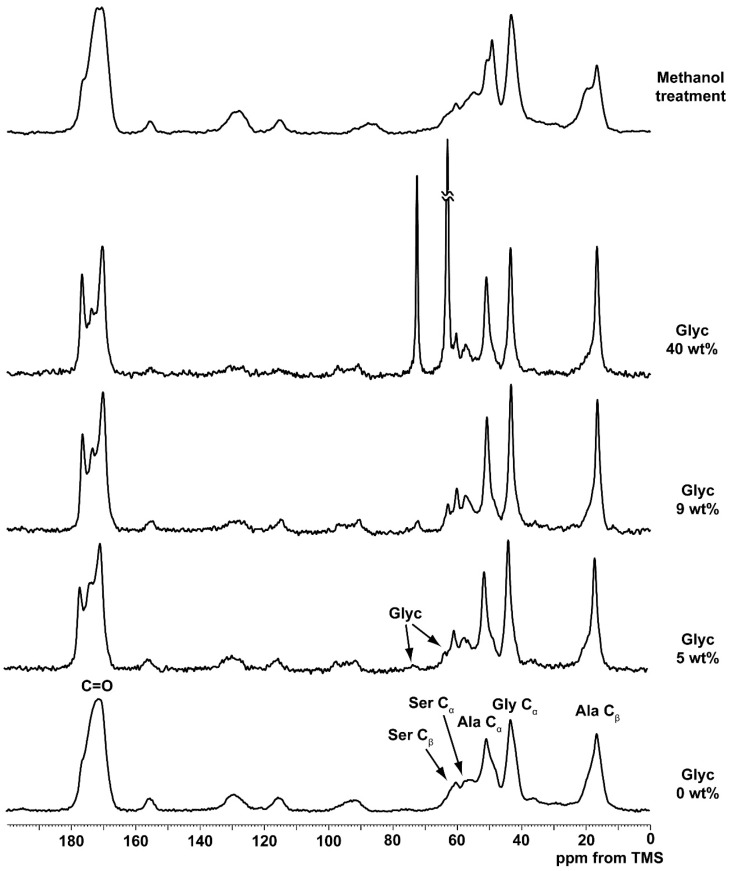
^13^C Cross Polarization/Magic Angle Spinning nuclear magnetic resonance (CP/MAS NMR) spectra of pure silk fibroin (SF) and glycerin (Glyc)-blend SF films with different Glyc concentrations of 5, 9, and 40 wt % and pure SF film treated by methanol. The assignments are given on top of the peaks. TMS, tetramethylsilane.

Figure 1 shows ^13^C CP/MAS NMR spectra of pure SF and Glyc-blended SF films with different Glyc concentrations of 5, 9, 40 wt % and pure SF film treated by methanol. Together with the peaks of SF, two small peaks assigned to Glyc were observed at 62.9 ppm (CH_2_) and 72.3 ppm (CH) even in 5 wt % Glyc-blended SF film. A further assignment of SF peaks to several conformations was performed with ^13^C conformation-dependent chemical shifts [13,14,17,20,21,22,33]. The ^13^C chemical shifts of random coil, Silk II and Silk I of Glyc-blended SF films are summarized in Table 1 together with ^1^H chemical shift data [32].

Without Glyc, the conformation of regenerated SF film was roughly random coil according to the Ala C_β_ chemical shift of 16.5 ppm, although there was a significant amount of β-sheet structure as mentioned below. By adding 5 wt % Glyc to SF, sharp C_β_ Ala (16.5 ppm) and C=O (177.0 ppm) peaks were newly observed together with Ser C_β_ (60.7 ppm) peak [12,13,33], indicating the partial generation of Silk I* form. At least 5 wt % Glyc concentration was enough to produce Silk I* form in SF through the strong interaction between SF and Glyc molecules in the dry state. The sample preparations of the Glyc-blended SF films and their NMR observations were repeated at least two times and confirmed the results.

Here, we start from the definition of Silk I* form is different from the Silk I structure; the details have been reported elsewhere [12,34]. Briefly, Silk I is defined as the solid state structure of SF stored in the middle silk glands after drying without any external forces. It is a soluble form that remains stable and non-viscous up to high concentrations without precipitating, this presumably being essential for the secretion of mature silk fibers [6,35,36]. According to solid state NMR spectra, the solid state Silk I contains random coil regions, together with regions having a well-defined ordered structure [13,14,33,34]. These ordered regions are defined as Silk I* [12,34]. Silk I* comes from the amino acid residues with the sequence (AGSGAG)_n_. However, it is important to point out that not all of the (AGSGAG)_n_ residues form Silk I*. A detailed recent analysis of ^13^C solid state NMR spectra of ^13^C selectively labeled SF [34] indicated that only longer (AGSGAG)_n_ regions contribute to Silk I*. This is entirely consistent with the hypothesis that Silk I* acts as a nucleus for the formation of Silk II structure during spinning of the silk fiber.

In this work, we aimed to interpret the structure of the Silk I* form in SF. The Silk I* is a repeated β-turn type II structure which was proposed to give the torsion angles, (φ, ψ) = (−62°, 125°) for Ala residues and (φ, ψ) = (77°, 10°) for Gly residues of poly(Ala-Gly) chain, thereby satisfying both solid state NMR and X-ray diffraction data. (The unit cell was orthorhombic and the space group was P2_1_2_1_2_1_, and the lattice constants were *a* = 4.65 Å, *b* = 14.24 Å and *c* = 8.88 Å, α = β = γ = 90°) [20,21,32]. The intra- and inter-molecular hydrogen bonding was formed alternatively along the chain. As noted earlier, the Silk I* form of longer (AGSGAG)_n_ sequences appeared as a result of the interaction between SF and Glyc molecules. A structural model for the complex of Glyc-SF having Silk I* form will be shown in Section 3.6.

Lu et al. [9] claimed the appearance of α-helix conformation in SF induced by the interaction between Glyc and SF molecules on the basis of Infrared spectroscopy (IR) analysis. However, from the results of NMR work mentioned above, it is clear that the newly appeared conformation was the Silk I* form, not α-helix. Many researchers other than Lu et al. in the field of SF research also reported the appearance of α-helix in SF from IR or Raman data of SF using automated analysis carried out with commercial software (for example, Opus 6.5 software, Bruker Optics Corp., Billerica, MA, USA). If there are poly(Ala) sequences in *B. mori* SF (as in the case of a wild-type silkworm, *Samia cynthia ricini* [11,35]), the sequences are expected to form α-helix. However, there are no poly(Ala) sequences in *B. mori* SF [37]. If the Ala residue forms the α-helical structure together with other amino acid residues, the Ala C_β_ peak should appear at 15 ppm in the ^13^C NMR spectrum. (It is known from the ^13^C conformation-dependent chemical shifts empirically and theoretically that the ^13^C chemical shifts of *the* amino acid residues reflect the secondary structure in the vicinity of the residues [11,20,21,22,23]). However, Ala C_β_ peak in this case appeared at 16.5 ppm for Silk I* form and not at 15 ppm. In addition, α-helix was clearly absent by comparing the observed 2D spin-diffusion NMR spectral patterns of (AG)_6_A[1-^13^C]G^14^[1-^13^C]A^15^G(AG)_7_ and (AG)_7_[1-^13^C]A^15^[1-^13^C]G^16^(AG)_7_ for the determinations of the torsion angles Ala^15^(φ, ψ) and Gly^14^(φ, ψ) in (AG)_15_, respectively, with the calculated patterns assuming the α-helix structure [21]. Indeed, Percot et al. had pointed out that discrimination between regular (α) and disordered (β-turn) helical conformations would be difficult from the Raman data [38,39]. In addition, the circular dichroism (CD) study of the concentrated SF in the middle silk gland of *B. mori* silkworm also gave α-helix-like structure [40,41]. We believe this confusion comes from the “special” structure of the Silk I* form. In our view, a theoretical approach involving IR, Raman and CD spectral patterns in view of the atomic coordinates of poly(Ala-Gly) with the repeated type II β-turn structure should give a solution to this problem.

As shown in Figure 1, at 9 wt % Glyc concentration the fraction of Silk I* increased slightly as evidenced by the intensity increase of the C=O (177.0 ppm) carbon peak. With further increase of Glyc concentration, the spectral change was very small as shown in the ^13^C CP/MAS NMR spectrum of Glyc(40 wt %)-blended SF film. These spectral patterns were quite different from the ^13^C CP/MAS NMR spectrum of SF film treated by methanol, which showed a typical Silk II form [13,14,30,31].

### 2.2. Quantitative Conformational Analysis of SF and Glyc-Blended SF from the Ala C_β_ Peaks of the ^13^C CP/MAS NMR Spectra

**Figure 2:**
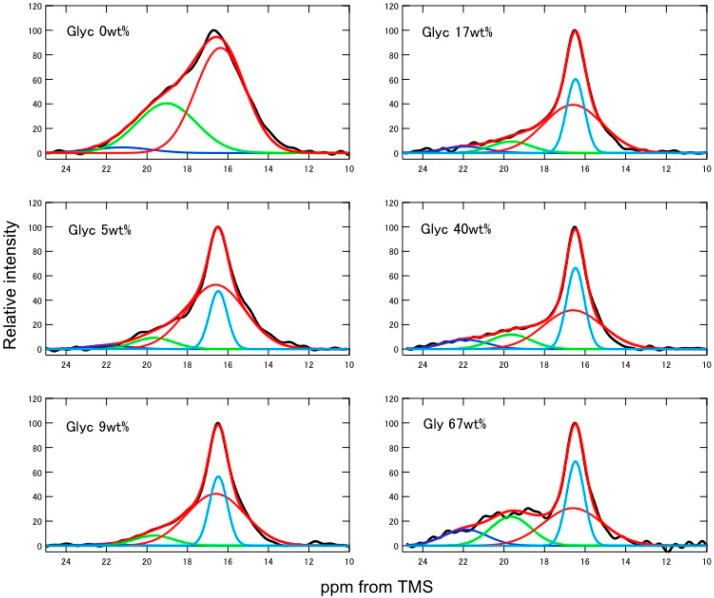
Deconvolution of Ala C_β_ peaks (marked by **red** (random coil), **light blue** (Silk I*), **dark blue** (β-sheet B) and **green** (β-sheet A) lines) in the ^13^C CP/MAS NMR spectra of SF and Glyc-blended SF films as a function of Glyc concentration by assuming Gaussian line shape. TMS, tetramethylsilane.

In order to determine the fraction of different conformations of SF and Glyc-blended SF films, deconvolution of the ^13^C Ala C_β_ peaks was performed as a function of Glyc concentration by assuming Gaussian line-shapes (Figure 2). Without Glyc, there were three components in the deconvoluted spectrum, i.e., random coil and two kinds of β-sheets, A and B. The β-sheet A and B were reported previously by us [12,22]. The torsion angles of both structures are the same (−140°, 140°) for both Ala and Gly residues. The β-sheet A and B have similar inter-molecular packing of the β-strands in the unit cell (*a* = 9.38 Å, *b* = 9.49 Å, *c* = 6.98 Å, and space group P2_1_) as reported by Takahashi et al. [42]. A key difference between β-sheet A and B is that the Ala methyl groups are positioned differently. In the β-sheet A, the methyl groups of the top sheet that point down to the central sheet are positioned roughly towards the Gly H_α_, in the spaces between the pairs of inter-strand Gly···Ala hydrogen bonds. In contrast, in the β-sheet B the methyl groups point to the center of the pair of inter-strand Gly···Ala hydrogen bonds and are thus shifted along the strand by one residue. In fact, the β-sheet A entailed slightly lower energy than the β-sheet B according to two structural models of (Ala-Gly)_15_ [22]. In the observed NMR spectrum of SF film alone, the β-sheet A was the main structure found on the basis of the chemical shifts.

During the preparation of the regenerated SF films (including the drying process), partial conformational change from random coil to β-sheet occurred, especially in the crystalline domain, which consisted of repeated AGSGAG sequences as reported previously [34]. By adding a small amount of Glyc (only 5 wt %) to SF, a remarkable change in the spectrum occurred. In particular, Silk I* form appeared partly as marked by light blue curve (Figure 2), viz. the peak with the narrower linewidth but the same chemical shifts as that of broad random coil peak. Thus, the Silk I* structure has a narrower chemical shift distribution than that of the random coil. In addition, β-sheet A in the spectrum of the SF sample without Glyc decreased considerably in intensity.

**Figure 3:**
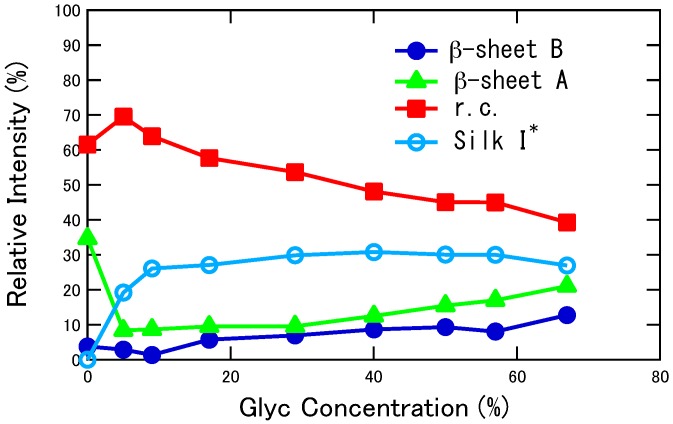
Change in the fraction of different conformations of SF and Glyc-blended SF films determined from the deconvolution of Ala C_β_ peaks as a function of Glyc concentration. r.c.: random coil.

The proportion of each conformation was determined by assuming the presence of only four conformations: Silk I*, random coil and β-sheets A and B. The change in the fraction of different conformations of SF and Glyc-blended SF films as a function of Glyc concentration is shown in Figure 3. The numerical values of the fractions are listed in Table S1. As Glyc concentration increased from 5 to 9 wt %, the change in the spectrum was not large compared with the spectral change from Glyc 0 to 5 wt %, but the fraction of Silk I* increased and that of random coil decreased. With further increase of Glyc content, the changes were relatively small. Thus, the fraction of Silk I* increased linearly up to 25%, then to 30% where it stayed almost constant. This was the same value (30%) of Ala residues in all Ala residues in SF sample present in *B. mori* mature silkworm. Thus, the fraction of 30% was considered to be the maximum content for Silk I* because only longer (AGSGAG)_n_ sequence could generate Silk I* form as discussed in our previous paper [34]. Thus, the minimum amount of Glyc to fully produce the Silk I* form was 9 wt %, and further Glyc addition did not generate more Silk I* structure in SF. With further increase of Glyc from 9 wt %, the fraction of random coil decreased and both β-sheets, A and B, increased gradually. Note that the amount of β-sheet A was larger than that of B over the whole range of Glyc concentrations.

### 2.3. ^1^H Solution NMR Spectra of Regenerated SF Aqueous Solution as a Function of Glyc Concentration

**Figure 4:**
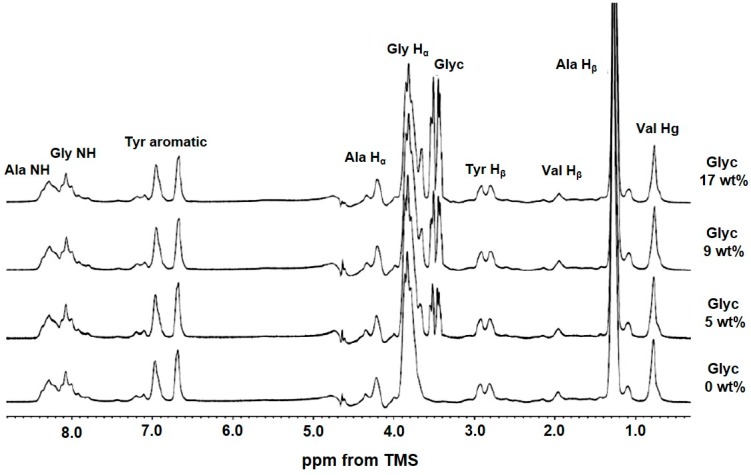
^1^H solution NMR spectra of regenerated SF aqueous solutions observed by changing Glyc concentration. The assignments are given on top of the peaks.

The ^1^H solution NMR spectra of regenerated SF aqueous solutions containing Glyc were observed as a function of Glyc concentration to study the interaction between SF and Glyc in aqueous solution (Figure 4). The NMR spectra were easily assigned by reference to a previous paper [36]. Other than SF peaks, the peaks assigned to Glyc were observed. However, with increasing Glyc concentration, there was no significant change. Thus, in aqueous solution, SF molecules were hydrated sufficiently and surrounded by water molecules. Similarly, Glyc molecules were also surrounded by sufficient amounts of water molecules. Therefore, there was essentially no direct interaction between SF and Glyc molecules. This indicated that the direct interaction between SF and Glyc occurred during the drying process because of the shortage of water. Thus only solid state NMR is useful for the purpose of structural characterization of SF and change in the structure as a function of Glyc concentration.

### 2.4. ^1^H Solid State NMR Spectra of SF and Glyc (29 wt %)-Blended SF Films

**Figure 5:**
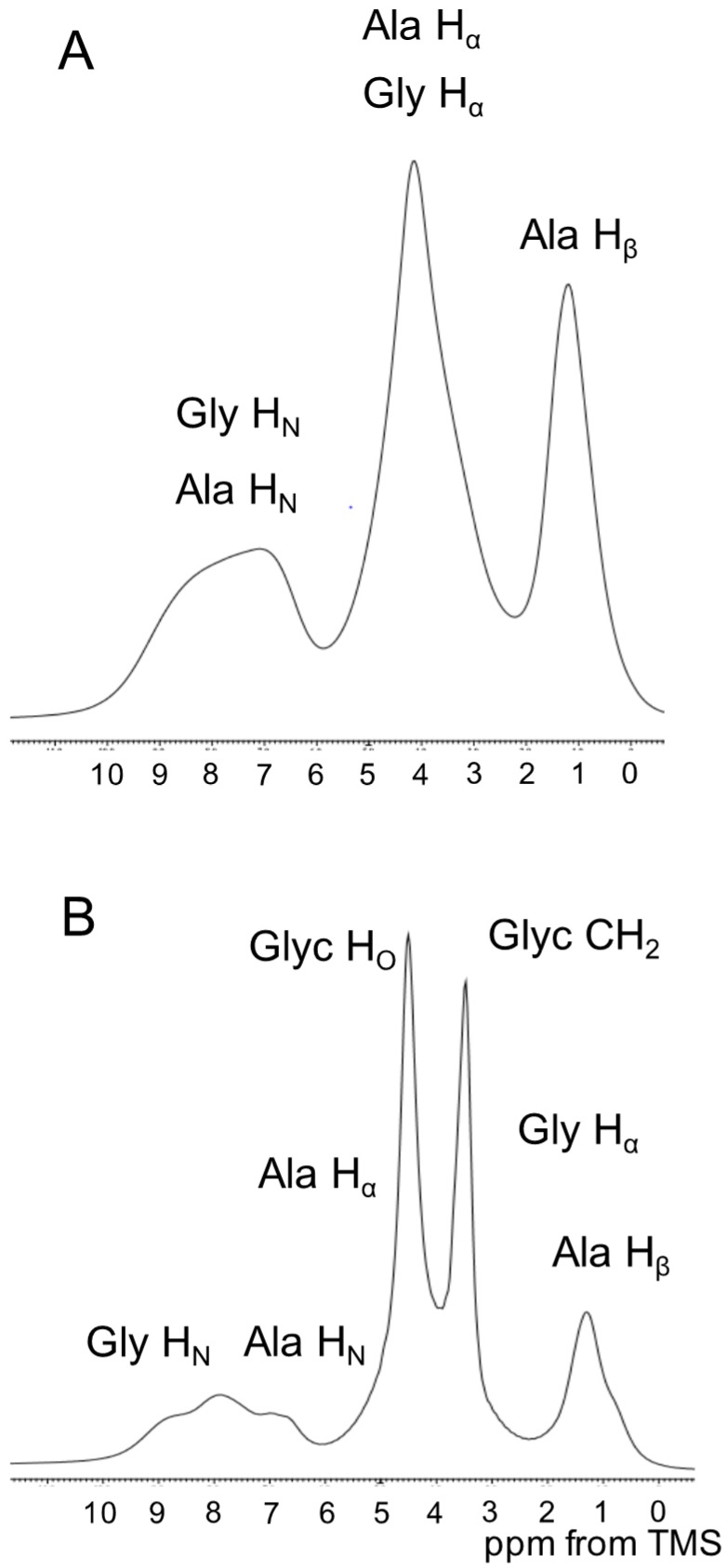
^1^H single pulse NMR spectra of (**A**) SF; and (**B**) Glyc (29 wt %)-blended SF films in the solid state. The assignments are given on top of the peaks. The detailed assignments and the chemical shifts of the ^1^H NMR peaks are summarized in Table 1 (^1^H chemical shift).

^1^H single pulse NMR spectra of SF and Glyc-blended SF films (Glyc 29 wt % concentration) were observed in the solid state (Figure 5). The Glyc (29 wt %)-blend SF film was selected because the fraction of Silk I* was fixed to be about 30%. There was a large difference in the spectrum between Glyc 0 wt % and Glyc 29 wt %. This was mainly due to the presence of Glyc peaks observed at 3.4 ppm (CH_2_ and CH) and 4.4 ppm (OH plus H_2_O) in the latter spectrum. In addition, there was a difference in the lower field (NH region) of the spectra. However, because of low resolution, the detailed assignments and related analysis was difficult, and further analysis was done from the ^1^H DQMAS NMR spectra (vide infra).

### 2.5. ^1^H Double-Quantum Magic Angle Spinning (DQMAS) NMR Spectrum of SF Film

**Figure 6:**
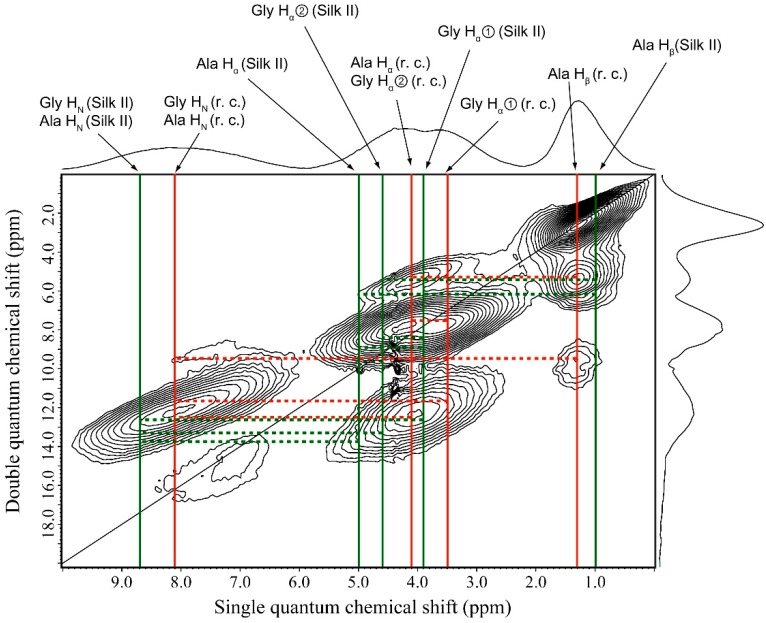
^1^H Double-Quantum Magic Angle Spinning (DQMAS) NMR spectrum of SF film together with the assignment. The ^1^H chemical shifts of random coil (**red**) and Silk II (**green**) forms are shown together with the ^1^H–^1^H correlation signals (broken lines).

The ^1^H DQMAS NMR spectrum of SF without Glyc is given in Figure 6. The fractions of random coil and β-sheet were determined to be 61.6% and 38.4%, respectively, from the simulation of the ^13^C CP/MAS NMR spectrum (Table S1). Thus, we need to consider the presence of these two conformations. In our previous paper [22], we reported ^1^H DQMAS NMR spectra of (AG)_15_ with Silk II form, which can serve as a reference spectrum for Silk II in the analysis of Figure 6. In our first attempt to assign the ^1^H DQMAS NMR spectrum, we compared the spectra of random coil with those of Silk II. Although differences in the chemical shifts between β-sheet A and B appeared in the ^13^C CP/MAS NMR spectra [22], it was difficult to distinguish the ^1^H peaks from the β-sheet A and B in the whole SF spectrum observed here. Therefore, we assume the chemical shift values of Silk II in Figure 6 correspond to the β-sheet A of (Ala-Gly)_15_.

The ^1^H chemical shifts of random coil and Silk II forms were determined as listed in Table 1. The most interesting points are the H_N_ chemical shifts which reflect the distance of direct hydrogen bonding of NH···OC pairs in the solid state [30] as well as the solution state [19]. The H_N_ chemical shifts of Ala and Gly residues with random coil were the same (8.1 ppm), but it was smaller than that of Silk II (8.7 ppm). The larger NH chemical shift indicated stronger inter-molecular hydrogen bond formation, and therefore the inter-molecular hydrogen bonding in Silk II was stronger than in random coil; this observation seemed to be reasonable. Ala H_β_ chemical shift of random coil was larger than that of Silk II, and Ala H_α_ chemical shift of Silk II was larger than that of random coil. This showed the same trend as ^1^H conformation-dependent chemical shifts of proteins [18,19]. As reported previously [22,27,32], the ^1^H DQMAS NMR spectrum gave information on the ^1^H–^1^H distances in the SF sample as observable cross peaks connecting two ^1^H nuclei within distances of about 4 Å. A set of six ^1^H–^1^H correlation signals (broken lines) was indicated in Figure 6 for SF with random coil form, while eight ^1^H–^1^H correlation signals for Silk II was found. The ^1^H–^1^H correlation data were summarized in Table 2. In view of the ^1^H–^1^H correlation data, both random coil and Silk II structure appeared present, although it was difficult to determine the fraction as in the case of ^13^C CP/MAS NMR as mentioned above.

### 2.6. ^1^H DQMAS NMR Spectrum of Glyc (29 wt %)-Blended SF Film

**Figure 7:**
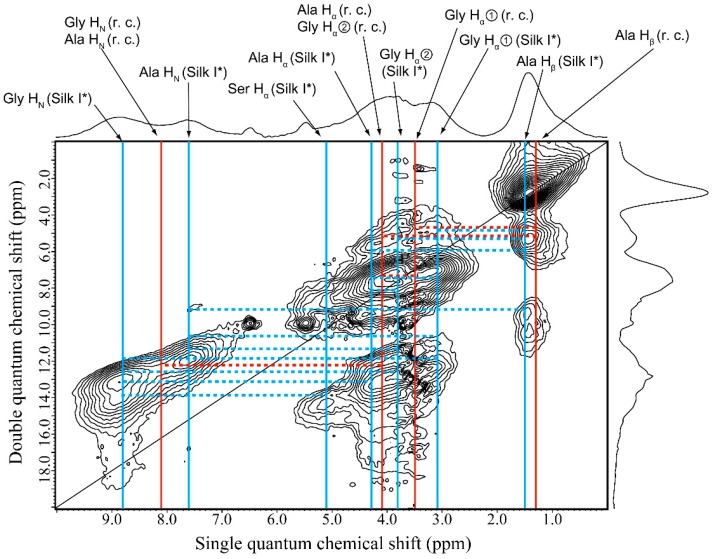
^1^H DQMAS NMR spectrum of Glyc (29 wt %)-blended SF film together with the assignments. The ^1^H chemical shifts of random coil (**red**) and Silk I* (**blue**) forms are shown together with the ^1^H–^1^H correlation signals (broken lines).

Figure 7 shows the ^1^H DQMAS NMR spectrum of SF with Glyc (29 wt %). The percentages of random coil, Silk I* and β-sheet were determined to be 53.6%, 29.9% and 17.5%, respectively, for Glyc (29 wt %)-blended SF. The remarkable spectral change from Figure 6 was due to the appearance of Silk I* form in SF. The Ser H_α_ peak of SF with Silk I* form was clearly observed in the ^1^H DQMAS NMR spectrum as well as the Ser C_α_ peak of SF with Silk I* form observed in the ^13^C CP/MAS NMR spectra of Glyc-blend SF films (Figure 1). In addition, the NH peaks of Ala and Gly residues were separated clearly with chemical shift difference of more than 1 ppm due to the appearance of Silk I* form [32]. In the Silk I* conformation, the Gly NH contributed to intra-molecular hydrogen bonding formation parallel to the SF chain, while Ala NH contributed to inter-molecular hydrogen bonding formation perpendicular to the SF chain [20,21]. The latter inter-molecular hydrogen bonding was weaker than the intra-molecular hydrogen bonding judging from the NH chemical shifts; thus, the NH chemical shifts of Ala H_N_ proton was 7.6 ppm and that of Gly H_N_ proton 8.8 ppm. Therefore, the inter-molecular hydrogen bonding was easy to break down by interaction with Glyc for the Silk I* form. A set of twelve ^1^H–^1^H correlation signals (broken lines) was observed for Silk I* form together with that of three ^1^H–^1^H correlation signals (broken lines) of random coil, as summarized in Table 2 although those of Silk II could not be detected because of low probability.

### 2.7. Structural Model of Glyc-SF Complex Having Silk I* Form

**Figure 8:**
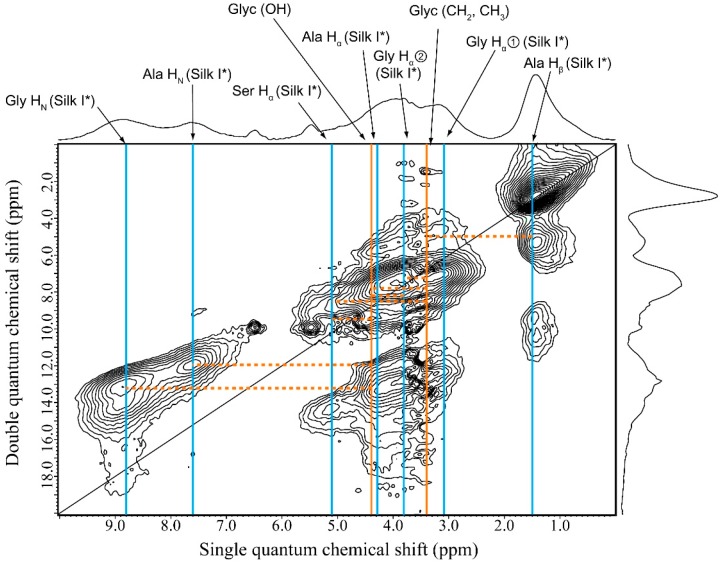
^1^H DQMAS NMR spectrum of Glyc (29 wt %)-blended SF film. The ^1^H–^1^H correlation signals (broken lines) between the OH or CH_2_ groups of Glyc (**orange**) and SF with Silk I* (**blue**) form are shown.

**Figure 9:**
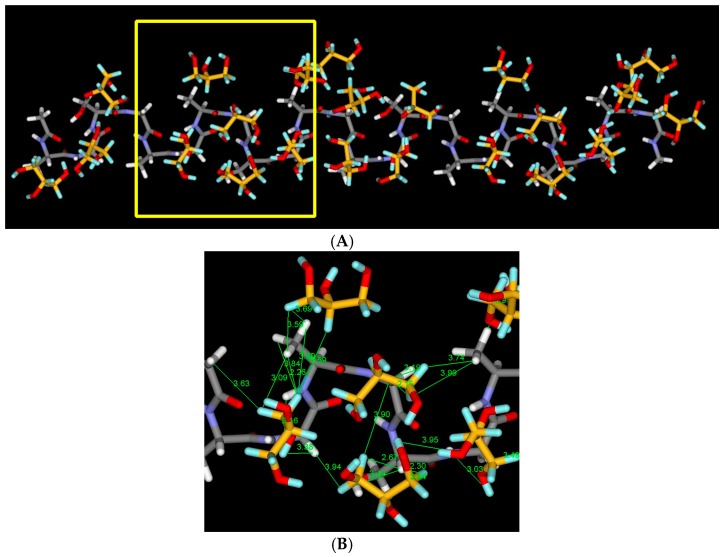
(**A**) A complex model of Glyc-SF model peptide, Acetyl-(Ala-Gly-Ala-Gly-Ser-Gly)_2_-NHCH_3_ with Silk I* form after 500 ps of Molecular Dynamics (MD) simulation. Details of the calculation are described in Materials and Method. The model satisfies the observed ^1^H–^1^H distance information. Four models including this model are shown in Figure S1; (**B**) the calculated distances between ^1^H atoms in Glyc and ^1^H atoms in SF in the area surrounded by square (**yellow**) in Figure 9A are shown as an example. All of the calculated ^1^H atomic distances between Glyc (CH_2_) and Ala H_β_, Glyc (CH_2_) and Gly H_α_①, Glyc (CH_2_) and Ser H_α_, Glyc (OH) and Ser H_α_, Glyc (OH) and AlaHN, and Glyc (OH) and Gly H_N_ are within 4 Å which satisfies the corresponding observed distances in Figure 8.

The six ^1^H–^1^H correlation signals (broken lines) between the OH or CH_2_ groups of Glyc and SF were selected from Figure 8 and listed in Table 2. Thus, ^1^H atomic distances of Glyc (CH_2_)-Ala H_β_, Glyc (CH_2_)-Gly H_α_①, Glyc (CH_2_)-Ser H_α_, Glyc (OH)-Ser H_α_, Glyc (OH)-Ala H_N_ and Glyc (OH)-Gly H_N_ were assumed to be within 4 Å. Here the Glyc peaks were observed at 3.4 ppm (CH_2_) and 4.4 ppm (OH plus H_2_O). The observed signals reflecting the distance constraints can be used to prepare a structural model for the Glyc-SF complex. As described in the section on Materials and Method, four complex models were obtained after MD simulation as shown in Figure S1. Figure 9A shows one example of the models, and the yellow highlighted area is expanded in Figure 9B to visualize the ^1^H–^1^H distances. The Glyc molecules are also hydrogen bonded with each other after the MD simulation. All the green lines in Figure 9B are within 4 Å, which satisfies the observed ^1^H–^1^H distance constraints in Glyc (29 wt %) -blended SF film. Among the four models in Figure S1, it is difficult to select one best model. Therefore, it seems reasonable to consider all these models to have similar probabilities. The complex between Glyc and SF with Silk I* form is very stable because the Silk I* form in Glyc-blended SF film does not decrease in concentration after immersion of the Glyc-blended SF film in methanol (data not shown).

## 3. Materials and Methods

### 3.1. Preparation of Glyc-Blended SF Films

The 25 cocoons from *B. mori* were degummed in a mixture of sodium carbonate (0.25% *w*/*v*) and Marseille soap (0.25% *w*/*v*) solution at 85 °C for 15 min in order to remove silk sericin [43]. Following this step, the degummed SF fiber was obtained. The SF fiber was then dissolved in 9 M LiBr aqueous solution at 40 °C. The 4% regenerated SF solution was prepared by dialysis of the 9 M LiBr aqueous solution against distilled water, followed by centrifugation at 10,000 rpm. The SF aqueous solution after mixing with a certain amounts of Glyc was cast on Teflon plates at 20 °C to prepare the Glyc-blend SF film [44]. The Glyc concentration in SF-Glyc mixture was changed from 0 to 67 wt %. There is no significant difference visually in the appearance and through Scanning Electron Microscopy (SEM) observations among the Glyc-blended SF films with different Glyc concentrations.

### 3.2. ^13^C CP/MAS NMR of Glyc-Blended SF Films

^13^C CP/MAS NMR spectra of Glyc-blended SF films were acquired on a Bruker DSX-400 AVANCE spectrometer (Bruker Co., Billerica, MA, USA) at room temperature operating at 100.4 MHz, with a CP contact time of 1 ms, two pulse phase modulation (TPPM) decoupling, and magic angle spinning at 7 kHz. A total of 8192 scans was collected over a spectral width of 60 kHz, with a recycle delay of 3 s. The ^13^C NMR chemical shifts were calibrated indirectly through the methylene peak of adamantane observed at 28.8 ppm relative to tetramethylsilane (TMS) at 0 ppm. The ^13^C CP/MAS NMR observations were repeated at least two times for newly prepared Glyc-blended SF films with different Glyc concentrations and the reproducibility of the experimental results was confirmed.

### 3.3. Deconvolution Analysis of ^13^C CP/MAS NMR Spectra

The Ala C_β_ peak in the ^13^C CP/MAS NMR spectra of SF films was used for the deconvolution analysis to determine the fraction of each conformation. In our previous papers [22,45,46], the Ala C_β_ peak was deconvoluted by assuming the presence of five peaks. The Ala C_β_ peak in the ^13^C CP/MAS NMR spectrum of the precipitated crystalline fraction of SF after chymotrypsin cleavage (Cp fraction (56% of total SF)) was independently observed and deconvoluted to three peaks at 21.7 ppm (β-sheet B), 19.6 ppm (β-sheet A) and 16.5 ppm (distorted β-turn/random coil) [22,46]. The Ala C_β_ peak in the ^13^C CP/MAS NMR spectrum of the other soluble fraction (44%) was assigned to the non-crystalline fraction [46]. However, it was difficult to monitor the structural change as a function of Glyc concentration because the structural change was expected to occur at both crystalline and non-crystalline regions of SF film simultaneously. Therefore in this paper, we determined the fraction of the conformation of Glyc-blended SF films by assuming the presence of four conformations: Silk I* (16.5 ppm), random coil (16.5 ppm), β-sheet A (19.6 ppm) and β-sheet B (21.7 ppm) from the Ala C_β_ peaks in the ^13^C CP/MAS NMR spectra. Since the Ala C_β_ chemical shifts were the same between random coil and Silk I*, the large difference in the half-height-widths between them (Random coil: ~300 Hz and Silk I*: ~100 Hz) was used to determine each fraction in the peak deconvolution. In addition, the appearance of Silk I* could be confirmed by the appearance of sharp peak at 177 ppm in the Ala carbonyl carbon region as reported previously [33,34]. All the deconvolution analyses were performed by assuming Gaussian line shapes [34,47].

### 3.4. Solid State DQMAS ^1^H NMR

DQMAS ^1^H NMR spectra were observed at 920 MHz using a JEOL JNM-ECA920 spectrometer in Okazaki, Japan [48]. The ^1^H–X double resonance and ultra-high speed MAS probe are attached. The sample spinning speed was stabilized such that the spinning fluctuations were less than ±10 Hz at a spinning rate of 70 kHz. The temperature of the samples was estimated to be around 333 K at 70 kHz MAS. The ^1^H rf field strength of π/2 pulse (1.29 μs) was 194 kHz. The ^1^H chemical shift was referenced to the peak of silicon rubber and set to 0.12 ppm from TMS. The 2τ delay was 0.3 ms. The DQMAS spectra were obtained every 32 scans at each period in the DQ domain, and the recycle delay was 2 s. For the ^1^H DQMAS measurement, a Dipolar Homonuclear Homogeneous Hamiltonian Double-Quantum/Single-Quantum correlation experiment (DH_3_DQ-SQ) was employed [49].

### 3.5. ^1^H Solution NMR of Regenerated SF Aqueous Solution

^1^H solution NMR spectra of regenerated SF aqueous solution were observed as a function of Glyc concentration at room temperature by JEOL ECX-400 spectrometer (JEOL Co., Tokyo, Japan).

### 3.6. Model Building of Glyc-SF with Silk I* Form by Molecular Dynamics (MD) Simulation

The MD simulation was performed for the complex model between Glyc and SF with Silk I* form by using the “Discover” module in Materials Studio 4.1 (Accelrys Inc. Tokyo, Japan). A crystal which consisted of 24 SF molecules (with the arrangement such that 6 molecules were located within the sheet and 4 molecules placed inter-sheet) with the formula Acetyl-(Ala-Gly-Ala-Gly-Ser-Gly)_2_-NHCH_3_ with Silk I* form [32] was built for the MD simulation. Five hundred Glyc molecules were generated around the crystal. All of the MD simulations were performed using a pcff force field in vacuo, and temperature was controlled at 298 K. The MD simulations were performed by 500,000 steps up to 500 ps. After the simulation, 16 Glyc-SF complex models where several Glyc molecules attached to each SF molecule located at the surface of the crystal were obtained at 500 ps. Moreover, the energy minimization was performed again for the complex models using MOPAC (Molecular Orbital PACkage, Colorado Springs, CO, USA). The models were selected if all of the observed 6 ^1^H–^1^H distances between ^1^H atoms of Glyc and SF were within 4 A. Finally, four complex models were obtained as shown in the Supplementary Materials.

## 4. Conclusions

The Glyc-induced structural characterization of SF was performed with ^13^C CP/MAS NMR and ^1^H DQMAS NMR. The presence of only 5 wt % Glyc in the film induced a significant conformational change in SF where Silk I* (repeated type II β-turn and no α-helix) newly appeared. Upon further increase in Glyc concentration, the percentage of Silk I* increased linearly up to 9 wt % Glyc and then tended to be almost constant (30%). The appearance of Silk I* form was confirmed by the ^1^H DQMAS NMR spectrum of Glyc-blended SF film. The ^1^H–^1^H distance constraints among ^1^H atoms of Glyc and ^1^H atoms of SF were obtained from the ^1^H DQMAS NMR and used to build a structural model of the complex between Glyc and SF having Silk I* form by MD simulation.

## Figures and Tables

**Table 1:** ^13^C and ^1^H chemical shifts (in ppm from tetramethylsilane (TMS)) of silk fibroin (SF) with different conformations in glycerin (Glyc)-blended SF film. The assignments of the conformations were performed as shown in the references [13,14,17,20,21,22,33] for ^13^C nuclear magnetic resonance (NMR) and [32] for ^1^H NMR.

**^13^C Chemical Shift**								
**Conformation**	**Ala C_β_**	**Ala C_α_**		**Ala CO**		**Gly C_α_**	**Gly CO**	**Ser C_β_**
r.c.	16.7	50.0		175.5		42.6	171.1–171.5	-
Silk II	19.6(A), 21.7(B)	49.2		172.6		43.0	169.1	-
Silk I*	16.5	51.4		177.0		43.8	170.7	60.7
**^1^H Chemical Shift**								
**Conformation**	**Ala H_β_**	**Ala H_α_**	**Ala H_N_**		**Gly H_α_①**	**Gly H_α_②**	**Gly H_N_**	**Ser H_α_**
r.c.	1.3	4.1	8.1		3.5	4.1	8.1	-
Silk II	1.0	5.0	8.7		3.9	4.6	8.7	-
Silk I*	1.5	4.3	7.6		3.8	3.1	8.8	5.1

r.c.: random coil; Silk I*: Type II β-turn; Gly H_α_① and Gly H_α_②: Two protons of Gly C_α_H_2_ group with different chemical shifts in the solid state [32].

**Table 2:** Sets of ^1^H–^1^H correlation signals in the ^1^H DQMAS NMR spectra of SF and Glyc (29 wt %)-blended SF films. These ^1^H–^1^H correlation signals are shown as broken lines in Figure 6, Figure 7 and Figure 8.

**SF Film**			
**r.c.**		**Silk II**	
Ala H_β_—Ala H_α_/Gly H_α_②		Ala H_β_—Gly H_α_②	
Ala H_β_—Gly H_α_①		Ala H_β_—Ala H_α_	
Ala H_β_—Ala H_N_/Gly H_N_		Gly H_α_①—Gly H_α_②	
Gly H_α_①—Ala H_α_/Gly H_α_②		Gly H_α_①—Ala H_α_	
Gly H_α_①—Ala H_N_/Gly H_N_		Gly H_α_①—Gly H_N_/Ala H_N_	
Ala H_α_/Gly H_α_②—Ala H_N_/Gly H_N_		Ala H_α_—Gly H_α_②	
-		Gly H_α_②–Gly H_N_/Ala H_N_	
-		Ala H_α_—Gly H_N_/Ala H_N_	
**Glyc-Blend SF Film**			
**r.c.**	Silk I*		Glyc—Silk I*
Ala H_β_—Ala H_α_/Gly H_α_②	Ala H_β_—Gly H_α_②		Glyc (CH_2_)—Ala H_β_
Ala H_β_—Gly H_α_①	Ala H_β_—Gly H_α_①		Glyc (CH_2_)—Gly H_α_①
Gly H_α_①—Ala H_α_/Gly H_α_②	Ala H_β_—Ala H_α_		Glyc (CH_2_)—Ser H_α_
Ala H_α_—Ala H_N_/Gly H_N_	Ala H_β_—Ala H_N_		Glyc (OH)—Gly H_α_①
-	Gly H_α_②—Ala H_α_		Glyc (OH)—Ser H_α_
-	Gly H_α_②—Ala H_N_		Glyc (OH)—Ala H_N_
-	Gly H_α_②—Gly H_N_		Glyc (OH)—Gly H_N_
-	Gly H_α_①—Ala H_α_		-
-	Gly H_α_①—Ala H_N_		-
-	Gly H_α_①—Gly H_N_		-
-	Ser H_α_—Gly H_N_		-
-	Ala H_α_—Gly H_N_		-

## Supplementary Materials

Supplementary materials can be found at www.mdpi.com/1422-0067/17/9/1517/s1.

## References

[1] Asakura T., Miller T. (2014). Biotechnology of Silk.

[2] Vollrath F., Porter D. (2006). Spider silk as archetypal protein elastomer. Soft Matter.

[3] Altman G.H., Diaz F., Jakuba C., Calabro T., Horan R.L., Chen J., Lu H., Richmond J., Kaplan D.L. (2003). Silk-based biomaterials. Biomaterials.

[4] Gronau G., Krishnaji S.T., Kinahan M.E., Giesa T., Wong J.Y., Kaplan D.L., Buehler M.J. (2012). A review of combined experimental and computational procedures for assessing biopolymer structure-process-property relationships. Biomaterials.

[5] Koh L.D., Cheng Y., Teng C.P., Khin Y.W., Loh X.J., Tee S.Y., Low M., Ye E., Yu H.D., Zhang Y.W. (2015). Structures, mechanical properties and applications of silk fibroin materials. Prog. Polym. Sci..

[6] Asakura T., Kaplan D.L., Arutzen C.J. (1994). Silk Production and Processing. Encyclopedia of Agricultural Science.

[7] Cao Y., Wang B. (2009). Biodegradation of Silk Biomaterials. Int. J. Mol. Sci..

[8] Kawahara Y., Furukawa K., Yamamoto T. (2006). Self-Expansion Behavior of Silk Fibroin Film. Macromol. Mater. Eng..

[9] Lu S., Wang X., Lu Q., Zhang X., Kluge J.A., Uppal N., Omenetto F., Kaplan D.L. (2010). Insoluble and Flexible Silk Films Containing Glycerol. Biomacromolecules.

[10] Pei Y., Liu X., Liu S., Lu Q., Liu J., Kaplan D.L., Zhu H. (2015). A mild process to design silk scaffolds with reduced β-sheet structure and various topographies at the nanometer scale. Acta Biomater..

[11] Asakura T., Suzuki Y., Nakazawa Y., Holland G.P., Yarger J.L. (2013). Elucidating silk structure using solid-state NMR. Soft Matter.

[12] Asakura T., Okushita K., Williamson M.P. (2015). Analysis of the Structure of *Bombyx mori* Silk Fibroin by NMR. Macromolecules.

[13] Asakura T., Kuzuhara A., Tabeta R., Saito H. (1985). Conformational characterization of *Bombyx mori* silk fibroin in the solid state by high-frequency carbon-13 cross polarization-magic angle spinning NMR, X-ray diffraction, and infrared spectroscopy. Macromolecules.

[14] Saito H., Tabeta R., Asakura T., Iwanaga Y., Shoji A., Ozaki T., Ando I. (1984). High-resolution carbon-13 NMR study of silk fibroin in the solid state by the cross-polarization-magic angle spinning method. Conformational characterization of silk I and silk II type forms of *Bombyx mori* fibroin by the conformation-dependent carbon-13 chemical shifts. Macromolecules.

[15] Van Beek J.D., Beaulieu L., Schäfer H., Demura M., Asakura T., Meier B.H. (2000). Solid-state NMR determination of the secondary structure of *Samia cynthia ricini* silk. Nature.

[16] Spera S., Bax A. (1991). Empirical correlation between protein backbone conformation and C_α_ and C_β_
^13^C nuclear magnetic resonance chemical shifts. J. Am. Chem. Soc..

[17] Asakura T., Iwadate M., Demura M., Williamson M.P. (1999). Structural analysis of silk with ^13^C NMR chemical shift contour plots. Int. J. Biol. Macromol..

[18] Wishart D.S., Sykes B.D., Richards F.M. (1991). Relationship between nuclear magnetic resonance chemical shift and protein secondary structure. J. Mol. Biol..

[19] Asakura T., Taoka K., Demura M., Williamson M.P. (1995). The relationship between amide proton chemical shifts and secondary structure in proteins. J. Biomol. NMR.

[20] Asakura T., Ashida J., Yamane T., Kameda T., Nakazawa Y., Ohgo K., Komatsu K. (2001). A repeated β-turn structure in poly(Ala-Gly) as a model for silk I of *Bombyx mori* silk fibroin studied with two-dimensional spin-diffusion NMR under off magic angle spinning and rotational echo double resonance. J. Mol. Biol..

[21] Asakura T., Ohgo K., Komatsu K., Kanenari M., Okuyama K. (2005). Refinement of Repeated β-turn Structure for Silk I Conformation of *Bombyx mori* Silk Fibroin Using ^13^C Solid-State NMR and X-ray Diffraction Methods. Macromolecules.

[22] Asakura T., Ohata T., Kametani S., Okushita K., Yazawa K., Nishiyama Y., Nishimura K., Aoki A., Suzuki F., Kaji H. (2015). Intermolecular Packing in *B. mori* Silk Fibroin: Multinuclear NMR Study of the Model Peptide (Ala-Gly)_15_ Defines a Heterogeneous Antiparallel Antipolar Mode of Assembly in the Silk II Form. Macromolecules.

[23] Jenkins J.E., Creager M.S., Lewis R.V., Holland G.P., Yarger J.L. (2010). Quantitative Correlation between the Protein Primary Sequences and Secondary Structures in Spider Dragline Silks. Biomacromolecules.

[24] Schnell I., Brown S.P., Low H.Y., Ishida H., Spiess H.W. (1998). An Investigation of Hydrogen Bonding in Benzoxazine Dimers by Fast Magic-Angle Spinning and Double-Quantum ^1^H NMR Spectroscopy. J. Am. Chem. Soc..

[25] Yates J.R., Pham T.N., Pickard C.J., Mauri F., Amado A.M., Gil A.M., Brown S.P. (2005). An Investigation of Weak CH···O Hydrogen Bonds in Maltose Anomers by a Combination of Calculation and Experimental Solid-State NMR Spectroscopy. J. Am. Chem. Soc..

[26] Yates J.R., Pickard C.J., Mauri F. (2007). Calculation of NMR chemical shifts for extended systems using ultrasoft pseudopotentials. Phys. Rev. B.

[27] Brown S.P. (2007). Probing proton–proton proximities in the solid state. Prog. Nucl. Magn. Reson. Spectrosc..

[28] Bradley J.P., Tripon C., Filip C., Brown S.P. (2009). Determining relative proton–proton proximities from the build-up of two-dimensional correlation peaks in ^1^H double-quantum MAS NMR: Insight from multi-spin density-matrix simulations. Phys. Chem. Chem. Phys..

[29] Harris R.K., Hodgkinson P., Zorin V., Dumez J.N., Herrmann B.E., Emsley L., Salager E., Stein R.S. (2010). Computation and NMR crystallography of terbutaline sulfate. Magn. Reson. Chem..

[30] Yazawa K., Suzuki F., Nishiyama Y., Ohata T., Aoki A., Nishimura K., Kaji H., Shimizu T., Asakura T. (2012). Determination of accurate ^1^H positions of an alanine tripeptide with anti-parallel and parallel β-sheet structures by high resolution ^1^H solid state NMR and GIPAW chemical shift calculation. Chem. Commun..

[31] Asakura T., Yazawa K., Horiguchi K., Suzuki F., Nishiyama Y., Nishimura K., Kaji H. (2014). Difference in the structures of alanine tri- and tetra-peptides with antiparallel β-sheet assessed by X-ray diffraction, solid-state NMR and chemical shift calculations by GIPAW. Biopolymers.

[32] Asakura T., Suzuki Y., Yazawa K., Aoki A., Nishiyama Y., Nishimura K., Suzuki F., Kaji H. (2013). Determination of Accurate ^1^H Positions of (Ala-Gly)_n_ as a Sequential Peptide Model of *Bombyx mori* Silk Fibroin before Spinning (Silk I). Macromolecules.

[33] Asakura T., Demura M., Date T., Miyashita N., Ogawa K., Williamson M.P. (1996). NMR study of silk I structure of *Bombyx mori* silk fibroin with ^15^N- and ^13^C-NMR chemical shift contour plots. Biopolymers.

[34] Asakura T., Sato Y., Aoki A. (2015). Stretching-Induced Conformational Transition of the Crystalline and Noncrystalline Domains of ^13^C-Labeled *Bombyx mori* Silk Fibroin Monitored by Solid State NMR. Macromolecules.

[35] Asakura T., Suzuki H., Watanabe Y. (1983). Conformational characterization of silk fibroin in intact *Bombyx mori* and *Philosamia cynthia ricini* silkworms by carbon-13 NMR spectroscopy. Macromolecules.

[36] Suzuki Y., Yamazaki T., Aoki A., Shindo H., Asakura T. (2014). NMR Study of the Structures of Repeated Sequences, GAGXGA (X = S, Y, V), in *Bombyx mori* Liquid Silk. Biomacromolecules.

[37] Zhou C.Z., Confalonieri F., Jacquet M., Perasso R., Li Z.G., Janin J. (2001). Silk fibroin: Structural implications of a remarkable amino acid sequence. Proteins.

[38] Percot A., Colomban P., Paris C., Dinh H.M., Wojcieszak M., Mauchamp B. (2014). Water dependent structural changes of silk from *Bombyx mori* gland to fibre as evidenced by Raman and IR spectroscopies. Vib. Spectrosc..

[39] Colomban P., Dinh H.M., Riand J., Prinsloo L.C., Mauchamp B. (2008). Nanomechanics of single silkworm and spider fibres: A Raman and micro-mechanical in situ study of the conformation change with stress. J. Raman Spectrosc..

[40] Asakura T. (1986). Structure of *Bombyx mori* silk fibroin in aqueous solution. Makromol. Chem. Rapid Commun..

[41] Asakura T., Ashida J., Yamane T., Cheng H.N., English A.D. (2004). Structure of *Bombyx mori* Silk Fibroin before Spinning in Silkworm. NMR Spectroscopy of Polymers in Solution and in the Solid State.

[42] Takahashi Y., Gehoh M., Yuzuriha K. (1999). Structure refinement and diffuse streak scattering of silk (*Bombyx mori*). Int. J. Biol. Macromol..

[43] Asakura T., Watanabe Y., Uchida A., Minagawa H. (1984). NMR of silk fibroin. Carbon-13 NMR study of the chain dynamics and solution structure of *Bombyx mori* silk fibroin. Macromolecules.

[44] Yoshimizu H., Asakura T. (1990). The structure of *Bombyx mori* silk fibroin membrane swollen by water studied with ESR, ^13^C-NMR, and FT-IR spectroscopies. J. Appl. Polym. Sci..

[45] Asakura T., Yao J., Yamane T., Umemura K., Ulrich A.S. (2002). Heterogeneous Structure of Silk Fibers from *Bombyx mori* Resolved by ^13^C Solid-State NMR Spectroscopy. J. Am. Chem. Soc..

[46] Asakura T., Yao J. (2002). ^13^C CP/MAS NMR study on structural heterogeneity in *Bombyx mori* silk fiber and their generation by stretching. Protein Sci..

[47] Asakura T., Isobe K., Aoki A., Kametani S. (2015). Conformation of Crystalline and Noncrystalline Domains of [3–^13^C]Ala-, [3–^13^C]Ser-, and [3–^13^C]Tyr-*Bombyx mori* Silk Fibroin in a Hydrated State Studied with ^13^C DD/MAS NMR. Macromolecules.

[48] Yamauchi K., Yamasaki S., Takahashi R., Asakura T. (2010). Microscopic structural analysis of fractured silk fibers from *Bombyx mori* and *Samia cynthia ricini* using ^13^C CP/MAS NMR with a 1 mm microcoil MAS NMR probehead. Solid State Nucl. Mag..

[49] Deschamps M., Fayon F., Cadars S., Rollet A.L., Massiot D. (2011). ^1^H and ^19^F ultra-fast MAS double-quantum single-quantum NMR correlation experiments using three-spin terms of the dipolar homonuclear Hamiltonian. Phys. Chem. Chem. Phys..

