# Supplementary material for: Glycerin-Induced Conformational Changes in Bombyx mori Silk Fibroin Film Monitored by 13C CP/MAS NMR and 1H DQMAS NMR
Source: Int J Mol Sci. 2016 Sep 9;17(9):1517. doi: 10.3390/ijms17091517 (PMC5037794; doi:10.3390/ijms17091517)
Supplement: Supplementary file 1 [file ijms-17-01517-s001.pdf]

Supplementary Materials: Glycerin-Induced Conformational Changes in *Bombyx mori* Silk Fibroin Film Monitored by ^{13}C CP/MAS NMR and ^{1}H DQMAS NMR

Tetsuo Asakura, Masanori Endo, Misaki Hirayama, Yuki Arai, Akihiro Aoki and Yugo Tasei

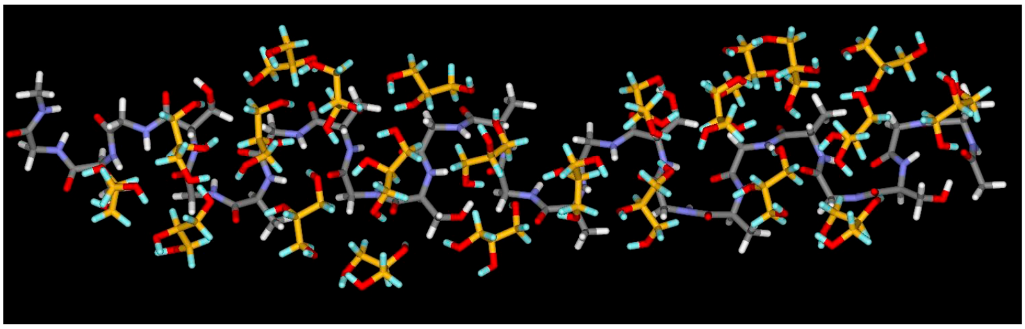

(**A**)

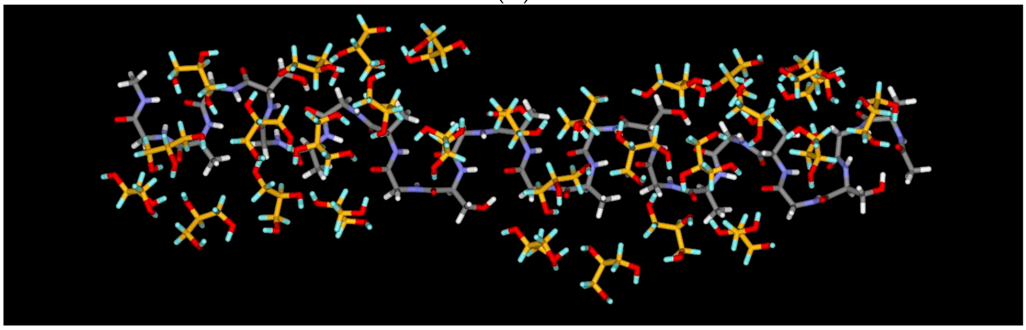

(**B**)

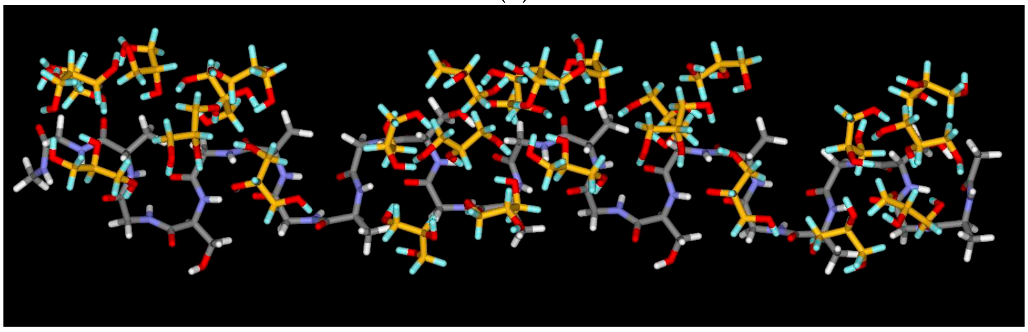

(**C**)

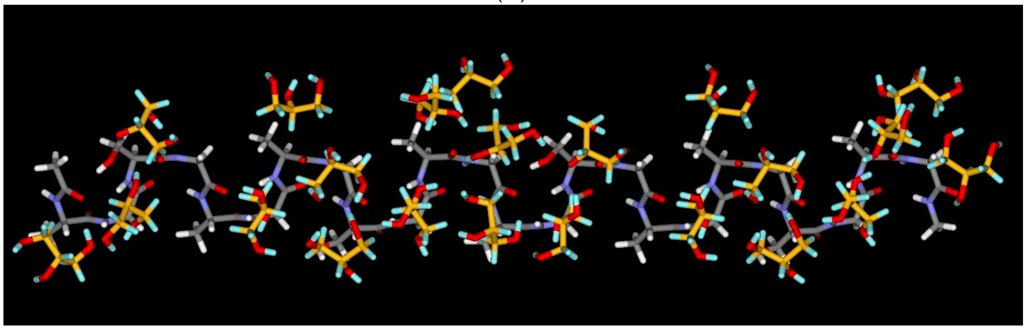

(**D**)

Figure S1. Four complex models, (**A–D**) of Glyc-SF model peptide, Acetyl-(Ala-Gly-Ala-Gly-Ser-Gly)$_2$-NHCH$_3$ with Silk I* forms after 500 ps of Molecular Dynamics (MD) simulations. Details of the calculation are described in Materials and Method.

Table S1. Fraction of several conformations determined from deconvolution of Ala Cβ peaks in ^{13}C Cross Polarization/Magic Angle Spinning nuclear magnetic resonance (CP/MAS NMR) spectra of silk fibroin (SF) and Glyc-blend SF films as a function of Glyc concentration.

Glyc (wt %)	β-Sheet B (%)	β-Sheet A (%)	r.c. (%)	Silk I* (%)
0	3.8	34.7	61.5	0.00
5	2.9	8.4	69.5	19.2
9	1.4	8.6	63.9	26.1
17	5.7	9.5	57.7	27.1
29	6.9	9.6	53.6	29.9
40	8.6	12.5	48.1	30.8
50	9.3	15.5	45.1	30.1
57	8.0	17.0	45.0	30.0
67	12.8	21.0	39.2	27.0

r.c.: random coil.
